# Binocular Astigmatism

**Published:** 1888-07

**Authors:** S. O. Richey

**Affiliations:** Washington, D. C.


### BINOCULAR ASTIGMATISM (?)

BY S. O. RICHEY, M. D.
Washington, D. C.

In a paper read in the Section on Ophthalmology, of the American Medical Association, by Dr. H. Culbertson, which is to be found in *The American Journal of Ophthalmology*, vol. v, 1888, p. 118, we may read as follows: "I have not infrequently encountered cases of astigmatism, in which, after having corrected the error in each eye separately, and on testing both eyes simultaneously in binocular vision, have found that vision proximum was not perfect, and, in order to attain normal vision near at hand in binocular sight, the angle denoting the axis of the cylindrical glass must be changed in one or both eyes. * * If the patient looks upon the floor, it will seem to incline to the right or left, and, on changing the axis of one or both cylinders, the surface will appear level."

The cases reported in illustration range, in age, from 18 to 34 years. He suggests the disturbance of the action of the extrinsic muscles in explanation.

This condition, which the writer calls "binocular astigmatism," is not uncommon, and, while I can recall no case in my experience so young as 18 years of age, many have come to my notice, after the age of presbyopia.

In such cases, vertical parallel lines appear to converge, generally toward the lower end, but often in the upward direction, and this remains true whether the object be near or at a distance. This peculiarity of vision with the correcting glasses is remarked as a curiosity; discomfort and annoyance are derived from the apparent slanting of the surface upon which he walks, one side of the pavement seeming to be higher than the other.

I have found, however, that even presbyopes, if they persist in wearing the cylindricals, as properly fitted with the aid of a mydriatic, will adjust themselves to the altered condition, and, with the lenses, find greatest comfort.

Dr. Culbertson's theory in explanation may be the correct one, but one of two others has seemed to me more satisfatory.

The relation of apparent *size* and *distance* is an individual experience. To a myope, all objects seen at all are larger and less clearly defined than to an emmetrope. The myope is accustomed to see only objects near to him with distinctness; therefore a distant object *is* near, and, when his myopia is first corrected, all objects seem to be nearer to him than is natural; the floor rises, and stairways are not exactly safe, because of this impression. To a hyperope, on the other hand, objects at a given distance appear smaller than to an emmetrope, and a + lens = H increases the apparent size of everything, and the distance therefore seems increased.

When either a myope, or a hyperope has worn a correcting glass long and constantly enough for adjustment he loses these false impressions and has comfortable vision. The occasional and judicious use of homatropine shortens greatly this period in hyperopes, astigmatics, and aniso-metropes; and for this reason, I have concluded that the cause of the difficulty might be found in the accommodation.

The experience of myopes, however, has led me to suspect a more central locus.

To illustrate; if an astigmatic sees a level floor inclining from right to left, an experience of years teaching him all the while that it *is* level, he finally accepts as a fact that all level surfaces *appear* to incline from right to left, and his mind habitually and unconsciously makes allowance for this. After he has acquired this habit he is enabled, by a correcting glass, to see level surfaces as they actually exist. As his cerebration has, in this particular, been unconscious, he continues unconsciously to make allowance, and the level pavement *appears* to incline from left to right proportionately. This habit is in time corrected by experience, under the new and true conditions.

This explanation seems to me to be the true one, but it is possible, though not perfectly clear to me, that the power of accommodation may contribute to this effect, because of our unconscious striving for definition of images.

The influence of the extrinsic muscles must be slight.

I beg to offer a recent case in illustration. March 27, 1888, Senator ———, æt. 60. For the past ten days he has had an inflammation of his lids. He gives no history of a former attack, but an examination discloses a blepharitis marginalis, and numerous inflamed and hypertrophied papillæ on the conjuntival surface of both upper and lower lids. Two of the hypertrophied papillæ (one on the right upper and the other on the right lower lid) suppurated and were opened on the conjunctival surface.

With each eye, $V = \frac{20}{L}$.

Under full mydriasis,

$$V.\ R. = \frac{20}{L} : \text{with} - 1.\ D\ s.\ \ V.\ R. = \frac{20}{XX}.$$

$$V.\ L. = \frac{20}{C} : \text{with} - 2.\ D\ cy.\ @90°,\ V.\ L. = \frac{20}{XX}.$$

After recovery from the effect of the mydriatic, with the use of the correcting glasses, vertical parallel lines appeared to converge from above downward. After a few weeks' use of the lenses, the distortion had grown markedly less, and, as usual, has probably disappeared. As he would not wear lenses on the street, I heard no complaint of the surface of the pavement; but, from numerous other experiences, I have no doubt it would have given the accustomed impression.

It is my practice, after assuring myself of the correctness of the lenses, to insist upon their constant use for a time, with the certainty that the ocular, or mental, readjustment, whichever it is, will take place.

Until some other, and more conclusive, evidence is offered, adverse to the theory of a mental process, this is to be preferred to one based upon the supposed inco-ordination of the extrinsic muscles, because any change of habit in muscular action is a result only of a very long process of training. On the contrary, the brain is capable of, and accustomed to, allowing for sudden changes in position of the body, and promptly adapts itself to its surroundings, in order to maintain control.

